# Retention of older veterans with serious mental illness in a clinical exercise program

**DOI:** 10.3389/fpsyt.2023.1221030

**Published:** 2023-06-22

**Authors:** Julia Browne, Eric B. Elbogen, Kim T. Mueser, James L. Rudolph, Wen Chih Wu, Noah S. Philip, Whitney L. Mills, Alexander S. Young, Richard Sloane, Katherine S. Hall

**Affiliations:** ^1^Center of Innovation in Long Term Services and Supports, VA Providence Healthcare System, Providence, RI, United States; ^2^Department of Psychiatry and Human Behavior, Alpert Medical School of Brown University, Providence, RI, United States; ^3^Geriatric Research, Education and Clinical Center, Durham VA Health Care System, Durham, NC, United States; ^4^Durham VA Health Care System, Durham, NC, United States; ^5^Department of Psychiatry and Behavioral Sciences, Duke University School of Medicine, Durham, NC, United States; ^6^Center for Psychiatric Rehabilitation and Departments of Occupational Therapy, Psychology, and Psychiatry, Boston University, Boston, MA, United States; ^7^Department of Health Services, Policy & Practice, Brown University, Providence, RI, United States; ^8^Medical Service, VA Providence Healthcare System, Providence, RI, United States; ^9^VA RR&D Center for Neurorestoration and Neurotechnology, VA Providence Healthcare System, Providence, RI, United States; ^10^Mental Illness Research, Education and Clinical Center, VA Greater Los Angeles, Los Angeles, CA, United States; ^11^Department of Psychiatry and Biobehavioral Sciences, University of California, Los Angeles, Los Angeles, CA, United States; ^12^Center for the Study of Aging and Human Development, Duke University School of Medicine, Durham, NC, United States

**Keywords:** physical activity, dropout, engagement, schizophrenia, bipolar disorder, recurrent major depressive disorder

## Abstract

Older adults with serious mental illness (SMI) have compromised physical function that could be improved with exercise; however, retention in exercise programs is a challenge. This study was a retrospective analysis of retention for the 150 older veterans with SMI that enrolled in Gerofit, a clinical exercise program offered in the Veterans Health Administration. Chi-square and *t*-tests were conducted to evaluate baseline differences between those that were and were not retained at six and 12 months. Retention was 33% and better health-related quality of life and endurance were related to retention. Future work is needed to improve exercise program retention in this population.

## 1. Introduction

Older people with serious mental illness (SMI) have mental and physical health challenges that reduce their functioning and quality of life (QOL) (1, 2). The physical functional decline in this group includes compromised endurance, strength, and mobility (3), which predict disability and mortality (4, 5). Exercise improves physical function in older adults (6) and in people with SMI (7); however, retention remains a major challenge for the SMI population (8).

Mental health symptoms, low motivation, medical concerns, medication side effects, and social isolation are obstacles to exercise engagement for people with SMI (9, 10). Disparities in access to quality care, which disproportionately affect Black adults (11), further exacerbate the health burden of SMI (12) and contribute to low participation in exercise and lifestyle programs (13–15). To address these barriers, many exercise and lifestyle programs for this group are offered at accessible locations (e.g., mental health clinics), include motivational components, and provide opportunities for social support (16, 17). Yet, most work has focused on young and middle-aged adults within research studies, and there is a lack of information on retention of older people with SMI in real-world exercise programs.

The purpose of this study was to examine retention of older veterans with SMI in Gerofit, an effective outpatient clinical exercise program available to eligible older veterans enrolled in the Veterans Health Administration (VHA) (18–20). The study aimed to evaluate differences in demographic and baseline health characteristics between older veterans with SMI that were and were not retained at six- and 12-months post-enrollment. We hypothesized that race (White), age (younger), SMI diagnosis (recurrent major depressive disorder), and baseline health status (fewer medical comorbidities, better physical function, and health-related QOL) would be associated with retention.

## 2. Method

### 2.1. Study design and participants

This study was a retrospective analysis approved by Institutional Review Boards at the Durham and Providence VA Healthcare Systems.

Veterans are eligible for Gerofit if they (a) are aged 65 or older, (b) are medically stable, and (c) have their own transportation. They are not eligible if they (a) are unable to independently perform activities of daily living, (b) experience cognitive impairment preventing safe exercise, (c) have any medical conditions for which exercise is contraindicated (e.g., unstable angina, active proliferative diabetic retinopathy, oxygen dependence, frank incontinence, active open wounds), (d) have an active substance use disorder, (e) are experiencing homelessness, (f) are unable to successfully participate in a group setting, and (g) are experiencing behavioral concerns that impact group participation (18, 21).

### 2.2. Gerofit program

Gerofit is a supervised, outpatient clinical exercise program offered in the VHA free of cost (21) except that veterans must have their own transportation to participate. Enrolled veterans complete an initial performance-based assessment of their functional capacity. Results of this initial assessment guide the creation of an individualized exercise prescription that include movements targeting endurance, strength, and mobility. Veterans are encouraged to complete their prescription multiple times per week in a group-based setting. Trained exercise staff provide supervision at gym facilities during specific hours.

### 2.3. Measures

#### 2.3.1. Retention

Six- and 12-month retention was defined as completion of the Gerofit assessment at the specified timepoint. Assessment completion is an adequate indicator of retention because testing is a core component of the Gerofit program and guides the exercise prescription (22).

#### 2.3.2. Baseline variables

Demographics (age, sex, race, ethnicity, marital status), SMI diagnosis (schizophrenia, schizoaffective disorder, bipolar disorder, recurrent major depressive disorder), psychiatric medication prescriptions (antipsychotics, mood stabilizers, antidepressants), and medical comorbidities (count of 26 possible medical conditions) (23) were obtained from the VHA electronic health record for the year prior to Gerofit enrollment.

Performance-based physical function measures included the six-minute walk test (endurance), 30-s arm curls and chair stands (strength), and the 8-foot-up-and-go and 10-meter walk tests (mobility) (24–26). Health-related QOL was assessed with the Patient-Reported Outcomes Measurement Information System Global Health Short Form (PROMIS) (27), which includes a single-item indicator of general health and three subscales (mental health, physical health, and satisfaction with social roles). These measures are completed at the Gerofit assessment.

### 2.4. Procedure

VHA clinical providers refer veterans to Gerofit. Gerofit staff assess eligibility through chart review followed by a phone screening. Interested and eligible veterans are then scheduled for a baseline assessment completed at the health facility.

### 2.5. Data analysis

Analyses were conducted with SAS (Version 9.4). Differences in baseline variables between older veterans with SMI that were and were not retained at six and 12 months were assessed with independent sample *t*-tests for continuous and chi-square tests for categorical variables.

## 3. Results

### 3.1. Participants

The sample included 150 older veterans with SMI (out of 1,414 total older veterans) that enrolled in Gerofit between 2010 and 2019 across eight VA sites (two northeast, two southeast, four west). The sample was, on average, 70 years old (SD = 5.4), predominately male (91%), White (53%) or Black (43%), not Hispanic or Latinx (95%), and not married (57%). Sixty-one percent had three or more medical comorbidities, 75% had a recurrent major depressive disorder diagnosis, and 77% had an antidepressant medication prescription. Retention was 33% at six and 12 months.

### 3.2. Baseline differences by retention status

Older veterans with SMI who were retained at 6 months had better health-related QOL on the PROMIS: general health [*t* (122) = −2.29, *p* = 0.02], physical health [*t* (43) = −2.12*, p* = 0.04], mental health [*t* (43) = −3.61*, p* = 0.001], and satisfaction with social roles [*t* (42) = −3.52, *p* = 0.001] than those who were not retained. None of the other measures were significantly related to six-month retention (all *p*s > 0.05) (Table 1).

**Table 1:** Comparison of baseline characteristics between older Veterans with SMI that were retained versus not retained at six and 12 months in Gerofit.

Baseline variable	Six months			12 months		
	Retained (*n* = 50)	Not retained (*n* = 100)	*p*	Retained (*n* = 50)	Not retained (*n* = 100)	*p*
Demographic, health, and clinical						
Age, *M* (SD)	69.9 (5.2)	70.5 (5.7)	0.53	69.6 (4.8)	70.6 (5.9)	0.30
Sex = Male, *n* (%)	47 (94)	90 (90)	0.41	47 (94)	90 (90)	0.41
Race, *n* (%)	–	–	0.17	–	–	0.08
White	22 (44)	58 (58)	–	21 (42)	59 (59)	–
Black	27 (54)	38 (38)	–	28 (56)	37 (37)	–
Asian, American Indian or Alaska Native, Native Hawaiian or other Pacific Islander, declined to answer or unknown^a^	1 (2)	4 (4)	–	1 (2)	4 (4)	–
Ethnicity = Hispanic or Latinx^b^, *n* (%)	1 (2)	5 (5)	–	1 (2)	5 (5)	–
Marital Status = Married, *n* (%)	25 (50)	40 (40)	0.24	23 (46)	42 (42)	0.64
Elixhauser comorbidity score, *n* (%)	–	–	0.85	–	–	0.46
0	5 (10)	6 (6)	–	5 (10)	6 (6)	–
1–2	14 (28)	34 (34)	–	16 (32)	32 (32)	–
3+	31 (62)	60 (60)	–	29 (58)	62 (62)	–
SMI diagnosis = Recurrent major depressive disorder, *n* (%)	38 (76)	75 (75)	0.89	38 (76)	75 (75)	0.89
Psychiatric medication prescriptions, *n* (%)	–	–	–	–	–	–
Antipsychotic	11 (22)	23 (23)	0.89	10 (20)	24 (24)	0.58
Mood stabilizer	15 (30)	31 (31)	0.90	17 (34)	29 (29)	0.53
Antidepressant	40 (80)	76 (76)	0.58	39 (78)	77 (77)	0.89
Self-reported health-related quality of life						
PROMIS – General Health^c^, M (SD)	2.98 (0.8)	2.59 (0.9)	**0.02**	2.81 (0.9)	2.67 (0.9)	0.41
PROMIS – Physical Health^d^, M (SD)	3.53 (0.7)	2.95 (0.7)	**0.04**	3.44 (0.7)	2.99 (0.7)	0.13
PROMIS – Mental Health^d^, M (SD)	3.50 (0.8)	2.51 (0.7)	**0.001**	3.28 (1.1)	2.59 (0.7)	**0.03**
PROMIS – Satisfaction with Social Roles^d^, M (SD)	3.67 (0.9)	2.54 (0.8)	**0.001**	3.38 (1.1)	2.64 (0.9)	**0.049**
Performance-based physical function						
Chair stand (total completed)^e^	11.2 (5.1)	10.2 (4.6)	0.24	11.3 (5.1)	10.2 (4.6)	0.16
Arm curl (total completed)^f^	16.9 (4.9)	16.2 (6.2)	0.66	17.6 (5.2)	16.1 (6.1)	0.31
Six-minute walk test (yards)^e^	469.7 (165.7)	417 (156.1)	0.06	476.1 (163.5)	413.9 (156.1)	**0.03**
8-foot-up-and-go (seconds)^e^	7.93 (3.7)	8.82 (5.7)	0.32	7.92 (3.6)	8.83 (5.8)	0.32
10-meter walk (meters/s)^e^	1.06 (0.2)	1.02 (0.3)	0.38	1.06 (0.2)	1.03 (0.3)	0.43

SMI, serious mental illness; PROMIS, patient-reported outcomes measurement information system.

Forty-three veterans completed both the six- and 12 months assessments, seven veterans completed only the six- month but not the 12-month assessment, and seven veterans did not complete the six-month but completed the 12-month assessment.

Row mean score test was used to evaluate differences in Elixhauser comorbidity score. Bold indicates *p* < 0.05.

a Categories were combined due to low frequencies.

b Unable to calculate due to low frequency of Hispanic or Latinx Veterans.

c Due to missing data, sample size for 6 months is *n* = 40 (retained), *n* = 84 (not retained) and 12 months is *n* = 42 (retained), *n* = 82 (not retained).

d The entire PROMIS measure was only administered to a subset of the cohort. Therefore, sample sizes for all subscales except the single item general health are smaller. Specific sample sizes are given below.

Physical health: 6 months (retained: *n* = 9, not retained: *n* = 36), 12 months (retained: *n* = 8, not retained: *n* = 37).

Mental health: 6 months (retained: *n* = 9, not retained: *n* = 36), 12 months (retained: *n* = 8, not retained: *n* = 37).

Satisfaction with social roles: 6 months (retained: *n* = 9, not retained: *n* = 35), 12 months (retained: *n* = 8, not retained: *n* = 36). One item included in this subscale was missing at six and 12 months for one participant.

e Chair stand was missing for 2 participants in the not retained group at 6 and 12 months. Six-minute walk test, 8-foot-up-and-go, and 10-meter walk was missing for one participant in the retained and one participant in the not retained groups at 6 and 12 months.

f Sample size for arm curl was lower than other tests because it was added to the Gerofit physical performance battery in more recent years. Sample size for arm curl is *n* = 20 (retained at 6 and 12 months) and *n* = 63 (not retained at 6 and 12 months).

Older veterans with SMI who were retained at 12 months had better health-related QOL on the PROMIS mental health [*t* (43) = −2.25, *p* = 0.03] and satisfaction with social roles [*t* (42) = −2.03, *p* = 0.049] subscales than those that were not retained. Those who were retained had better endurance on the six-minute walk test [*t* (146) = −2.25, *p* = 0.03] than those who were not retained. None of the other measures were significantly related to 12-month retention (all *p*s > 0.05) (Table 1).

## 4. Discussion

This study examined retention of older veterans with SMI in a supervised exercise program. Retention was 33%; but 86% percent of veterans with SMI that were retained at six months remained in the program to 12 months. Better baseline health-related QOL and endurance were the only variables associated with retention. These findings highlight the need for additional tailoring of clinical exercise programming to better retain the older veteran population with SMI, particularly during the first six months and for those with lower initial QOL and endurance.

Our retention rate of 33% is much lower than those reported in large SMI health promotion trials (≥68%) (28, 29, 30). The present analysis focused on retention in a free-of-cost clinical exercise program without any monetary incentives and was not conducted as a research trial where dedicated outreach and compensation for assessments are often standard. Further, Gerofit is designed broadly for older veterans and does not include specific tailoring for the SMI population. Nonetheless, the low retention rate is surprising given that Gerofit does address many established barriers to exercise for the SMI population (e.g., social support, access, cost, supervision) (8, 9). These findings suggest that there are SMI-specific barriers to exercise engagement that are not adequately addressed in Gerofit, which may include, among others, unreliable transportation and low motivation (31).

Better health-related QOL and endurance were the only baseline characteristics that distinguished retention status. Prior research has also shown that better self-reported physical health but not specific medical conditions were related to greater exercise in SMI (32, 33). None of the demographic or mental health variables were related to retention. Homogeneity of our sample with respect to age and SMI diagnosis may have obscured potential relationships. But, the non-significant relationship between race and retention is consistent with prior work showing reduced racial disparities between Black and White older veterans with SMI in a VHA clinical program compared to other programs (34). As such, Gerofit may reduce racial disparities in retention through its cost-free availability to older veterans.

The sample comprised mainly male veterans although the racial diversity of Gerofit is a strength. Further, we did not have data on exercise attendance or adherence. Finally, aside from the general health single item, the PROMIS scale was only administered to a subset of participants. Future exercise programs for this population should consider alternate strategies to enhance retention in the SMI population. For example, home-based models of exercise are effective for older adults and improve engagement by limiting the need for transportation (35, 36). In fact, home-based programs that include motivational strategies have shown initial promise for those with SMI in improving engagement and targeting the well-established motivational difficulties present for this group (37). Therefore, future exercise programs developed for the older population with SMI should consider home-based or hybrid (i.e., combination home-based and facility-based) modalities in tandem with activities targeting motivation to improve retention. Overall, this study highlights an opportunity to modify existing clinical exercise programming for older veterans to address the unmet needs of the older SMI population.

## Data availability statement

The original contributions presented in the study are included in the article/supplementary material, further inquiries can be directed to the corresponding author.

## Ethics statement

The studies involving human participants were reviewed and approved by the Institutional Review Boards at Durham and Providence VA Healthcare Systems. Written informed consent for participation was not required for this study in accordance with the national legislation and the institutional requirements.

## Author contributions

JB, EE, KM, and KH conceptualized the study. RS conducted the data analyses. JB wrote the first draft. JR, WCW, NP, WM, and AY provided the feedback and input on the analytical interpretation and manuscript writing. All authors contributed to the article and approved the submitted version.

## Funding

This work was supported by a VA Rehabilitation Research and Development Career Development Award (IK1RX003904) to JB. The Durham Gerofit program has been locally supported by the Durham VA Geriatric, Research, Education and Clinical Program, and KH is supported in part by VA RR&D (RX003120), and the Duke Claude D. Pepper Older Americans Independence Center (NIA P30 AG028716), and the Duke Roybal Center (NIA P30 AG064201).

## Conflict of interest

The authors declare that the research was conducted in the absence of any commercial or financial relationships that could be construed as a potential conflict of interest.

## Publisher’s note

All claims expressed in this article are solely those of the authors and do not necessarily represent those of their affiliated organizations, or those of the publisher, the editors and the reviewers. Any product that may be evaluated in this article, or claim that may be made by its manufacturer, is not guaranteed or endorsed by the publisher.

## Author disclaimer

The views expressed in this article are those of the authors and do not necessarily reflect the position or policy of the United States Government or Department of Veterans Affairs.
